# Public Attitudes, Interests, and Concerns Regarding Polygenic Embryo Screening

**DOI:** 10.1001/jamanetworkopen.2024.10832

**Published:** 2024-05-14

**Authors:** Rémy A. Furrer, Dorit Barlevy, Stacey Pereira, Shai Carmi, Todd Lencz, Gabriel Lázaro-Muñoz

**Affiliations:** 1 Center for Bioethics, Harvard Medical School, Boston, Massachusetts; 2 Center for Medical Ethics and Health Policy, Baylor College of Medicine, Houston, Texas; 3 Braun School of Public Health and Community Medicine, The Hebrew University of Jerusalem, Jerusalem, Israel; 4 Institute of Behavioral Science, The Feinstein Institutes for Medical Research, Northwell Health, Manhasset, New York; 5 Departments of Psychiatry and Molecular Medicine, Zucker School of Medicine at Hofstra/Northwell, Hempstead, New York; 6 Division of Research, Department of Psychiatry, The Zucker Hillside Hospital Division of Northwell Health, Glen Oaks, New York; 7 Department of Psychiatry, Massachusetts General Hospital, Boston

## Abstract

**Importance:**

Polygenic embryo screening (PES) is a novel technology that estimates the likelihood of developing future conditions (eg, diabetes or depression) and traits (eg, height or cognitive ability) in human embryos, with the goal of selecting which embryos to use. Given its commercial availability and concerns raised by researchers, clinicians, bioethicists, and professional organizations, it is essential to inform key stakeholders and relevant policymakers about the public’s perspectives on this technology.

**Objective:**

To survey US adults to examine general attitudes, interests, and concerns regarding PES use.

**Design, Setting, and Participants:**

For this survey study, data were collected from 1 stratified sample and 1 nonprobability sample (samples 1 and 2, respectively) between March and July 2023. The surveys measured approval, interest, and concerns regarding various applications of PES. In the second sample, presentation of a list of potential concerns was randomized (presented at survey onset vs survey end). The survey was designed using Qualtrics and distributed to participants through Prolific, an online sampling firm. Sample 1 was nationally representative with respect to gender, age, and race and ethnicity; sample 2 was recruited without specific demographic criteria. Analyses were conducted between March 2023 and February 2024.

**Main Outcomes and Measures:**

Participants reported their approval, interest, and concerns regarding various applications of PES and outcomes screened (eg, traits and conditions). Statistical analysis was conducted using independent samples *t* tests and repeated-measures analyses of variance.

**Results:**

Of the 1435 respondents in sample 1, demographic data were available for 1427 (mean [SD] age, 45.8 [16.0] years; 724 women [50.7%]). Among these 1427 sample 1 respondents, 1027 (72.0%) expressed approval for PES and 1169 (81.9%) expressed some interest in using PES if already undergoing in vitro fertilization (IVF). Approval among these respondents for using PES for embryo selection was notably high for physical health conditions (1109 [77.7%]) and psychiatric health conditions (1028 [72.0%]). In contrast, there was minority approval for embryo selection based on PES for behavioral traits (514 [36.0%]) and physical traits (432 [30.3%]). Nevertheless, concerns about PES leading to false expectations and promoting eugenic practices were pronounced, with 787 of 1422 (55.3%) and 780 of 1423 (54.8%) respondents finding them very to extremely concerning, respectively. Sample 2 included 192 respondents (mean [SD] age 37.7 [12.2] years; 110 men [57.3%]). These respondents were presented concerns at survey onset (n = 95) vs survey end (n = 97), which was associated with less approval (28–percentage point decrease) and more uncertainty (24 percentage–point increase) but with only slightly higher disapproval (4 percentage–point increase).

**Conclusions and Relevance:**

These findings suggest that it is critical for health care professionals and medical societies to consider and understand the perspectives of diverse stakeholders (eg, patients undergoing IVF, clinicians, and the general public), given the absence of regulation and the recent commercial availability of PES.

## Key Points

**Question:**

Among US adults, what are the general attitudes, interests, and concerns regarding polygenic embryo screening (PES)?

**Findings:**

In this survey study exploring public sentiment toward PES, there was high approval and interest among respondents despite high societal concerns. In a second sample, presenting concerns at survey onset (vs survey end) was associated with less public approval and more uncertainty but with only slightly higher disapproval.

**Meaning:**

With the recent commercial availability of unregulated PES, the combination of high approval and high concerns observed among respondents in this study suggests the need for informed dialogue and guidance, particularly in addressing the divide between the public and health care professionals.

## Teaser

This survey study examines general attitudes, interests, and concerns among US adults regarding the use of polygenic embryo screening.

## Introduction

Polygenic embryo screening (PES), also known as preimplantation genetic testing for polygenic outcomes (PGT-P), is an emerging biotechnology currently used in the context of in vitro fertilization (IVF) to screen and rank individual embryos for their future genetic likelihood of developing common, complex health conditions. Until recently, preimplantation genetic testing was limited to monogenic conditions that occur due to single-gene variations (eg, sickle cell disease).^1^ However, with the advent of genome-wide association studies, polygenic scores have been developed for predicting physical health conditions (eg, coronary heart disease or type 1 diabetes),^2,3^ psychiatric health conditions (eg, schizophrenia or depression),^4,5^ behavioral traits (eg, educational attainment or extraversion),^6,7^ and physical traits (eg, height or skin color).^8,9^ Although studies demonstrate that applying polygenic scores to embryos is practically feasible,^10,11,12,13^ the technology faces substantial limitations due to the correlational nature of polygenic scores and to their low predictive power, their variable performance across environments and populations, and the limited genetic diversity between sibling embryos.^14,15,16,17,18,19,20,21^ Beyond these limitations, PES has raised psychosocial concerns among clinicians, patients, genetic counselors, bioethicists, psychologists, and lawyers due to its potential impact on individuals and society at large.^8,22,23,24,25,26,27,28,29,30,31,32^ Despite these limitations and concerns, PES is already commercially available in the US and unregulated as to the types of polygenic outcomes that can be screened.^33,34^

Companies offering PES presently limit their screening to physical and psychiatric health conditions. However, one company previously offered screening for some traits, which suggests that it could be offered again in the future. Thus, it is critical to understand how the public perceives the use of PES for health conditions and an array of traits. Given the recent emergence of this technology, only 2 short surveys have published results, and these have been limited to gauging US public approval and interest in PES for specific outcomes.^35,36^ In this study, we examined 24 conditions and traits, informed by qualitative interviews with US-based reproductive endocrinologists and infertility specialists (REIs) and patients undergoing IVF.^37^ We assessed both approval and interest for a variety of PES applications. To our knowledge, this is the first survey to examine public views on concerns regarding PES. Furthermore, in a second sample, we experimentally investigated the association between informing the public of potential concerns and their ratings of approval and interest in PES.

## Methods

This survey study was approved by the Baylor College of Medicine institutional review board. Informed consent was obtained when participants clicked the “agree to participate” option during the survey. The study followed the American Association for Public Opinion Research (AAPOR) reporting guideline.

### Participants

The survey was programmed in Qualtrics software (Qualtrics International Inc) and distributed through Prolific, an online sampling firm, to a nationally representative sample of US participants (stratified on gender, race and ethnicity, and age). Participants had to meet the following eligibility criteria: aged 18 to 100 years, US nationality, and currently residing in the US. Gender identity was self-reported. Information on race and ethnicity was collected to obtain opinions representative of the general US population and was categorized based on National Institutes of Health funding mandates. Race was self-reported as American Indian or Alaska Native, Asian, Black or African American (hereinafter, Black), Native Hawaiian or Pacific Islander, White, or other race (open-ended response option). Ethnicity was self-reported as Hispanic or Latino (hereinafter, Hispanic) or non-Hispanic.

Sample 1 participants were recruited from Prolific with nationally representative stratified sampling for gender, race and ethnicity, and age. Sample 2 participants also were recruited from Prolific but did not have a nationally representative quota for demographic characteristics. Participants randomly assigned to sample 2a were presented with the concerns first, whereas those assigned to sample 2b were presented with the concerns last (at the end of the survey). This study aimed to compare findings across the 2 experimental conditions, so there was less concern for representativeness.

### Survey Design

The design of items assessing potential uses of PES and information types along with potential concerns was informed by qualitative data collected from 53 interviews with US-based REIs and patients undergoing IVF.^37^ Survey preregistrations and materials are available in eMethods 1 and 2 in Supplement 1. Survey participants first received an animated introduction to PES, which briefly explained IVF, monogenic testing, and polygenic testing. They were then presented multiple embryo reports detailing the various percentiles, averages, and absolute risk estimates for several conditions for 2 embryos (see eMethods 3 in Supplement 1 for calculation of the risk estimates, which used small to medium plausible effect sizes^13^). Finally, participants were informed that PES provides only genetic estimates, that the environment and chance also play an important role in the development of conditions, and that current polygenic scores are not as accurate for people of non-European ancestries. We included 7 comprehension check questions that provided corrective feedback if answered incorrectly. As preregistered, participant data inclusion was restricted to those who answered a minimum of 5 of 7 comprehension questions correctly.

We asked participants about general approval of PES, how they think they would vote on allowing or disallowing PES, and their perceived risk to benefit ratio regarding people accessing this biotechnology. Subsequently, participants rated their approval of screening embryos for 12 conditions and 12 traits that were identified in the qualitative interviews.^37^ Participants were then presented with a 4 × 4 matrix and rated whether they approved, disapproved, or did not have an opinion for using PES for the following 4 purposes: information, preparation, embryo selection, and embryo selection based on family history. For each purpose, participants rated their approval for 4 screened outcomes: physical health conditions, psychiatric health conditions, behavioral traits, and physical traits. Participants also rated their interest in using PES (assuming they were undergoing IVF), their likelihood of undergoing IVF for the purpose of using PES (assuming they wanted a child), and their willingness to pay for PES (assuming the cost of IVF was covered). Participants then rated their level of concern for 13 potential concerns about PES (eg, false expectations or promoting eugenics), before finally reporting their demographic information.

For sample 1, we recruited 1535 participants. Of those who began the survey, 1491 completed it, and 1435 (96.2%) correctly answered 5 or more of the comprehension checks. For sample 2, 200 of 202 participants completed the full survey, and 192 (96.0%) passed 5 or more of the comprehension checks correctly. A power analysis suggested that 175 participants were needed to detect an effect size of Cohen *d* = 0.3 at *P* < .05 with 80% power. We collected data from 202 participants, anticipating exclusions for potential comprehension check failures.

### Statistical Analysis

As preregistered, we averaged across the 12 traits and 12 conditions (measured on 5-point Likert scales, with 1 indicating strongly disapprove and 5 indicating strongly approve) and conducted a 2-tailed, dependent samples *t* test to analyze mean differences in approval between traits and conditions. For the 4 × 4 matrix, responses were summed across outcomes and purposes to run 2 exploratory repeated-measures analyses of variance (ANOVAs). We conducted the Mauchly test of sphericity and applied Huynh-Feldt corrections. We also ran post hoc analyses with Bonferroni corrections to account for multiple comparisons.

We ran exploratory 2-tailed independent samples *t* tests. These tests were conducted to assess whether age (≤35 years vs >35 years), interest in having (additional) children, and number of children were associated with approval of PES.

Because the sample was stratified only on gender, age, and race and ethnicity, we examined the distributions of other demographic characteristics: political ideology, income, and education and conducted weighted analyses based on US nationally representative distributions. All analyses are included in eTables 1 to 3 in Supplement 1.

In sample 2 (n = 192), we experimentally randomized whether participants were asked to rate the 13 potential concerns first (at the start of the survey, following the introduction) or last (at the end of the survey, preceding the demographic questions). We preregistered the following hypotheses: participants in the concerns-first group will be less accepting, less interested, and more concerned than those in the concerns-last group. We used 2-tailed, independent samples *t* tests to analyze mean differences across groups. We conducted a series of 2-tailed, independent samples *t* tests to test for differences in approval, interest, and concerns between samples 1 and 2a because they completed the same survey (eAppendix in Supplement 1).

For all analyses, we used *P* < .05 (2-tailed) as the threshold for statistical significance. Statistical analyses were conducted in JASP, version 0.17.0 (Apple Silicon; JASP Team) and SPSS, version 28.01.1 (IBM SPSS). Analyses were conducted between March 2023 and February 2024.

## Results

### Participants

Demographic characteristics are detailed in the Table. For sample 1, demographic data were available for 1427 of the 1435 respondents (mean [SD] age, 45.8 [16.0] years). There were 724 individuals who identified as female (50.7%), 656 as male (46.0%), and 47 (3.3%) as another gender. A total of 16 respondents (1.1%) were American Indian or Alaska Native, 91 (6.3%) were Asian, 189 (13.2%) were Black, 2 (0.1%) were Native Hawaiian or Pacific Islander, 1150 (80.1%) were White, and 20 (1.4%) were of other race (participants could select multiple races; therefore, percentages in both samples sum to >100%). In terms of ethnicity, 81 respondents (5.7%) were Hispanic and 1346 (94.3%) were non-Hispanic.

**Table.** Demographic Characteristics of Respondents^a^

Characteristic	Sample 1 (n = 1427)	Sample 2		
		Total sample (n = 192)	2a, Concerns first (n = 95)	2b, Concerns last (n = 97)
Age, mean (SD), y	45.8 (16.0)	37.7 (12.2)	36.5 (11.3)	38.8 (12.9)
Gender				
Women	724 (50.7)	74 (38.5)	38 (40)	36 (37.1)
Men	656 (46.0)	110 (57.3)	51 (53.7)	59 (60.8)
Other^b^	47 (3.3)	8 (4.2)	6 (6.3)	2 (2.1)
Race^c^				
American Indian or Alaska Native	16 (1.1)	7 (3.6)	3 (3.2)	4 (4.1)
Asian	91 (6.3)	22 (11.3)	9 (9.5)	13 (13.4)
Black or African American	189 (13.2)	18 (9.4)	7 (7.4)	11 (11.3)
Native Hawaiian or Pacific Islander	2 (0.1)	1 (0.5)	1 (1.1)	0 (0.0)
White	1150 (80.1)	155 (80.7)	78 (82.1)	77 (79.4)
Other^d^	20 (1.4)	2 (1.0)	2 (2.1)	0 (0.0)
Ethnicity				
Hispanic or Latino	81 (5.7)	14 (7.3)	6 (6.3)	8 (8.2)
Non-Hispanic	1346 (94.3)	178 (92.7)	89 (93.7)	89 (91.8)
Education level				
Less than bachelor degree	642 (45.0)	94 (49.0)	41 (43.2)	53 (54.7)
Bachelor degree or higher	785 (55.0)	98 (51.0)	54 (56.8)	44 (45.3)
Household income, $				
0-49 999	596 (41.8)	81 (42.2)	34 (35.8)	47 (48.5)
≥50 000-109 999	553 (38.8)	77 (40.1)	44 (46.3)	33 (34.0)
≥110 000	277 (19.4)	34 (17.7)	17 (17.9)	17 (17.5)
Parent of a child				
No	707 (49.5)	122 (63.5)	61 (64.2)	61 (62.9)
Yes	728 (50.5)	70 (36.5)	34 (35.8)	36 (37.1)
Political orientation				
Conservative	361 (25.3)	43 (22.4)	24 (25.3)	19 (19.6)
Liberal	811 (56.9)	113 (58.9)	58 (61.1)	55 (56.7)
Moderate	254 (17.8)	36 (18.7)	13 (13.7)	23 (23.7)

^a^ Unless otherwise indicated, values are No. (%) of respondents.

^b^ Includes self-reported identities. For sample 1, respondents answered agender (n = 1), nonbinary (n = 2), transgender woman (n = 9), transgender man (n = 10), and gender queer or nonconforming (n = 26). For sample 2, responses included gender queer or nonconforming (n = 2).

^c^ In both samples, participants could select multiple races; therefore, percentages sum to greater than 100%.

^d^ Includes self-reported races. For sample 1, respondents answered Hispanic (n = 3); multiple, mixed (n = 2); human (n = 2); Mexican American, Latinx, mixed or human, Middle Eastern, Arabic Middle Eastern, or Jewish (n = 2); European American, Middle East and North Africa, Hebrew, biracial Afro-Caribbean American or White European American (n = 2); or category not defined (n = 1). For sample 2, respondents answered Colombiana, not defined (n = 1).

For sample 2, demographic data were available for 192 respondents (mean [SD] age, 37.7 [12.2] years). There were 74 individuals who identified as female (38.5%), 110 as male (57.3%), and 8 (4.2%) as another gender. A total of 7 respondents (3.6%) were American Indian or Alaska Native, 22 (11.3%) were Asian, 18 (9.4%) were Black, 1 (0.5%) was Native Hawaiian or Pacific Islander, 155 (80.7%) were White, and 2 (1.0%) were of other race. In terms of ethnicity, 14 respondents (7.3%) were Hispanic and 178 (92.7%) were non-Hispanic.

### Sample 1

#### Approval of PES Use

**Figure 1.**
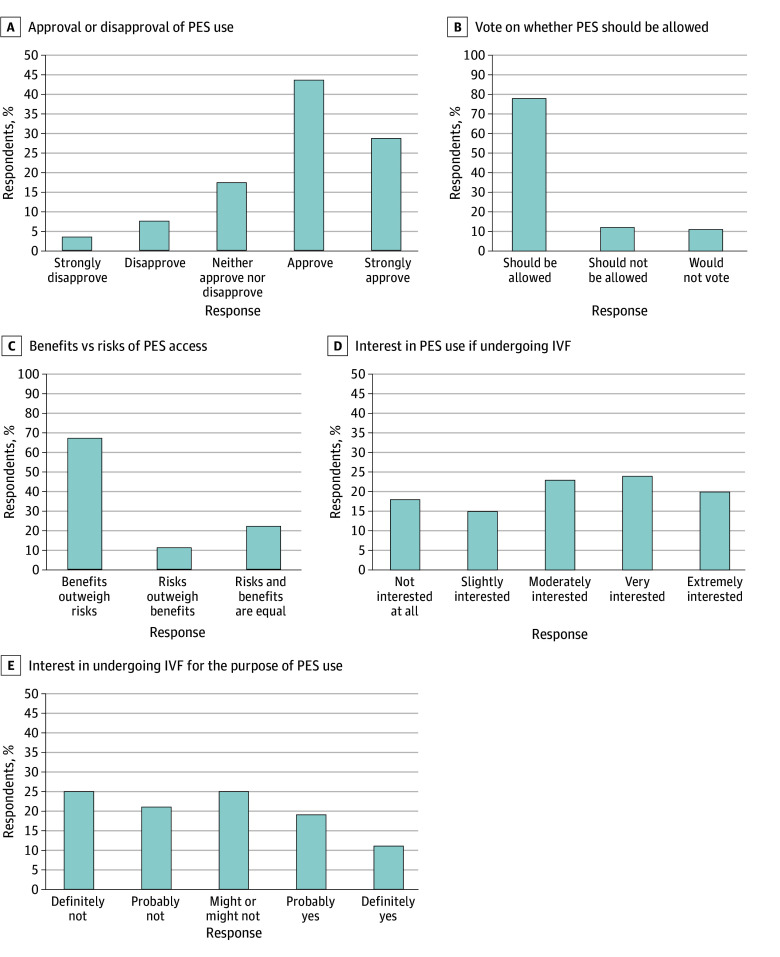
Approval and Interest in Polygenic Embryo Screening (PES) Respondents were asked the following questions: A, “Do you approve or disapprove of people using polygenic embryo screening?” B, “If you were to vote on whether polygenic embryo screening should be allowed, how would you vote?” C, “Which of these statements comes closest to your views about allowing people to have access to polygenic embryo screening: the benefits outweigh the risks, the risks outweigh the benefits, or the risks and benefits are equal?” D, “Suppose that you (or your partner) are undergoing in vitro fertilization (IVF). How interested would you be in using polygenic embryo screening?” E, “Suppose you were to have a child. Would you consider undergoing in vitro fertilization (IVF) for the specific purpose of using polygenic embryo screening?” The number of respondents for these items varied from 1427 to 1428. Percentages are rounded to the nearest whole number, which, if summed according to the figures, can lead to percentage points being greater than or less than 100%.

As depicted in the first Likert plot in Figure 1A, 1027 of 1427 respondents (72.0%) in sample 1 either approved or strongly approved of people using PES. Only 156 of 1427 respondents (10.9%) either disapproved or strongly disapproved, and 246 (17.2%) were ambivalent. These attitudes were similarly reflected in voting intentions: 1107 of 1428 respondents (77.5%) stated that they would vote to allow the use of PES, 155 (10.9%) would vote to disallow it, and 166 (11.6%) would abstain from voting (Figure 1B). When gauging respondent perceptions of the PES risk to benefit ratio, 958 of 1428 respondents (67.1%) believed that the benefits of allowing people to access PES outweigh the risks, 161 (11.3%) believed that the risks outweigh the benefits, and 309 (21.6%) believed the risks and benefits to be equivalent (Figure 1C).

#### Interest in PES Use

When participants were asked to gauge their interest in using PES under the circumstances of already undergoing IVF, 1169 of 1427 (81.9%) reported being at least slightly interested (Figure 1D). When asked whether participants would consider undergoing IVF for the specific purpose of getting access to PES, 427 of 1427 (29.9%) reported they would probably or definitely consider using it, 649 (45.5%) reported they would probably or definitely not consider using it, and 351 (24.6%) reported that they might or might not consider using it (Figure 1E). Participants who answered that they were at least slightly interested in PES (n = 1170) were also asked to report the maximum amount they would be willing to pay to access PES, with the assumption that the cost of IVF was covered. The responses ranged from $0 to $100 000, with the mean (SD) willingness to pay being $3240 ($815) and the median being $1000 (IQR, $2600).

#### Approval of PES for Purposes Across Outcomes

**Figure 2.**
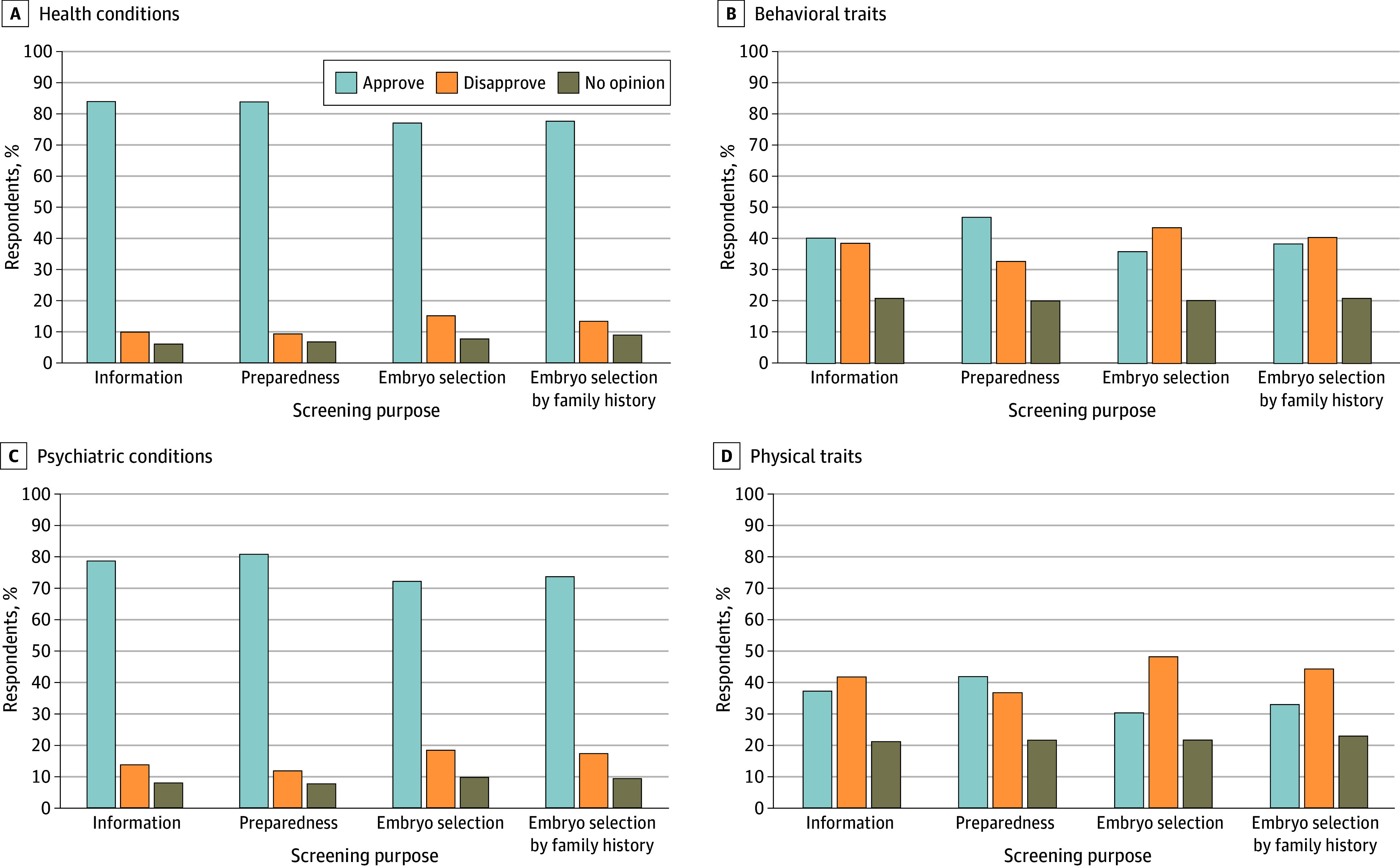
Approval of Polygenic Embryo Screening (PES) Use for Varying Purposes and Outcomes Participants (n = 1427) were asked to answer the following prompt: “I approve/disapprove of people using polygenic embryo screening for the following purposes based on the listed conditions and traits.” Responses are presented for screening for health conditions (A), behavioral traits (B), psychiatric conditions (C), and physical traits (D). Purposes were presented to participants as follows: information (people using PES to know more about their future child), preparation (people using PES to prepare [resources] for their future child), embryo selection (people using PES to select embryos to use with their preferred genetic chances), and embryo selection given family history (people using PES to select embryos to use with their preferred genetic chances for traits or conditions that run in their family).

Descriptive results of approval for different purposes across outcomes are depicted in Figure 2. Responses across the approval matrix of outcomes and purposes were coded numerically (1, approve; −1, disapprove; and 0, no opinion).

##### Purposes (Information or Selection)

Responses were summed across outcomes to test whether there were significant differences in approval between the following purposes: preparation (mean [SE], 1.63 [0.06]), information (mean [SE], 1.36 [0.06]), embryo selection based on family history (mean [SE], 1.07 [0.07]), and embryo selection (mean [SE], 0.90 [0.07]). The Mauchly test of sphericity was significant, which required the use of Huynh-Feldt corrections. A repeated-measures ANOVA demonstrated significant differences in approval across purposes (partial η^2^ = 0.07; *P* < .001). Post hoc tests using Bonferroni corrections revealed significant differences across all purposes (*P* < .001).

##### Outcomes (Traits and Conditions)

Responses were summed across purposes to test whether there were differences in approval between the following outcomes: physical health conditions (mean [SE], 2.75 [0.63]), psychiatric health conditions (mean [SE], 2.44 [0.70]), behavioral traits (mean [SE], 0.60 [0.85]), and physical traits (mean [SE], *−*0.29 [0.83]). The Mauchly test of sphericity was significant, which required the use of Huynh-Feldt corrections. A repeated-measures ANOVA demonstrated significant differences in approval across outcomes (partial η^2^ = 0.39; *P* < .001). Post hoc tests using Bonferroni corrections revealed significant differences across all outcomes (*P* < .001). Of 1427 respondents, most approved of PES use for embryo selecton based on physical health conditions (1101 [77.2%]) and psychiatric health conditions (1028 [72.0%]). However, a minority approved of embryo selection for behavioral traits (514 [36.0%]) and physical traits (432 [30.3%]). When the survey specified that the purpose of PES was for parents to prepare for their future child, approval among the 1427 respondents increased to 84.0% (n = 1198) for physical health conditions, 80.6% (n = 1150) for psychiatric health conditions, 47.0% (n = 671) for behavioral traits, and 41.8% (n = 597) for physical traits.

#### Approval of PES Use for Specific Conditions and Traits

**Figure 3.**
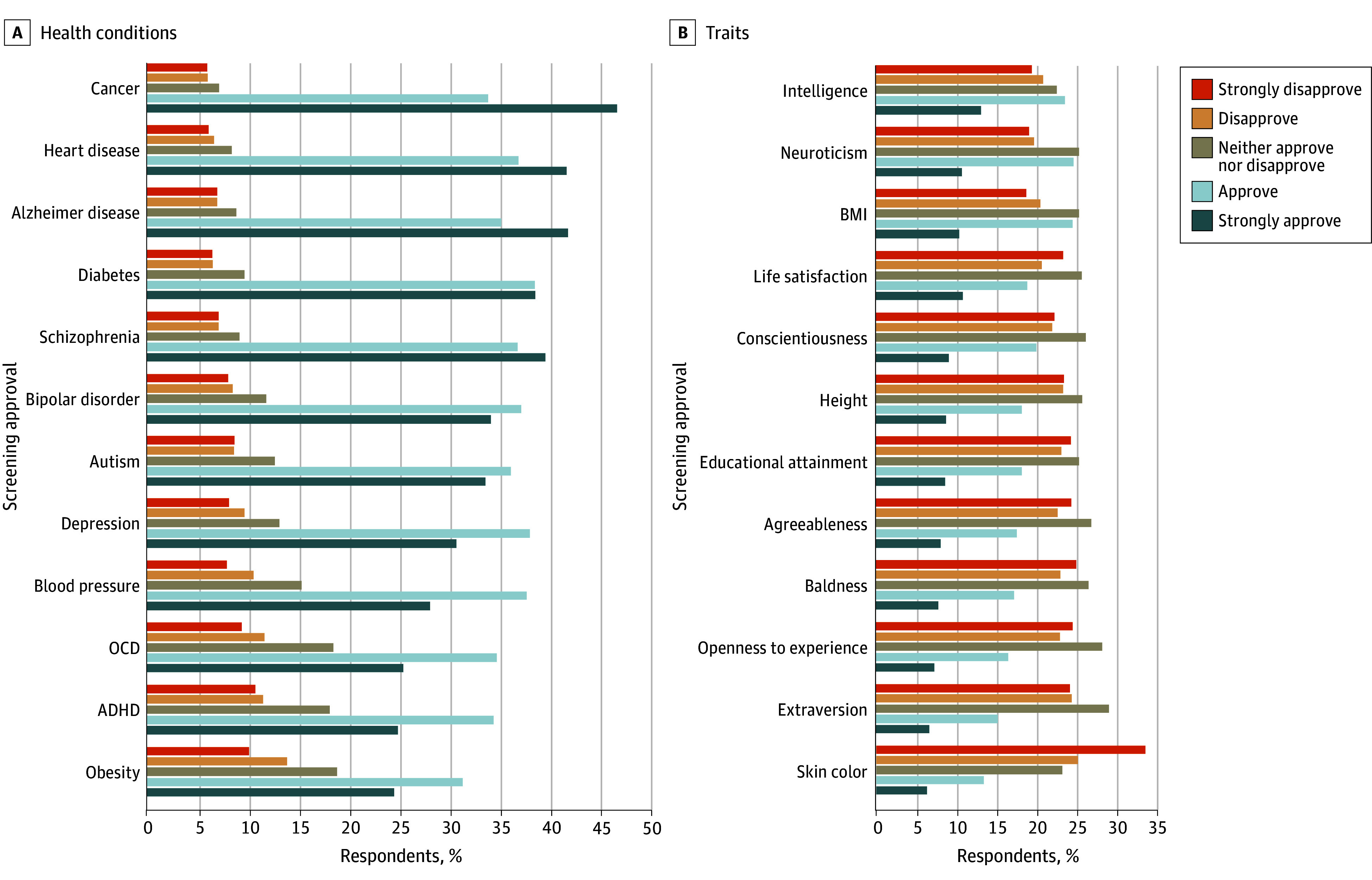
Approval of Polygenic Embryo Screening (PES) Use for Conditions and Traits Approval ratings for screening for health conditions (A) and traits (B) ranked by mean approval (in descending order). The number of respondents for these items was 1427, except there were 1428 respondents for depression and cancer. Percentages are rounded to the nearest whole number, which, if summed according to the figures, can lead to percentage points being greater than or less than 100%. ADHD indicates attention-deficit/hyperactivity disorder; BMI, body mass index (calculated as weight in kilograms divided by height in meters squared); OCD, obsessive-compulsive disorder.

We asked participants to rate their approval for screening embryos on 12 conditions (physical and psychiatric; Figure 3A) and 12 traits (behavioral and physical; Figure 3B). Physical health conditions received the highest approval for screening (eg, cancer: 1150 of 1426 [80.6%]; heart disease: 1122 of 1427 [78.6%]; and Alzheimer disease: 1100 of 1427 [77.1%]), with fewer than 15% of respondents (n = 173-200 of 1426-1427) disapproving of screening for those conditions. Schizophrenia received the highest screening approval of the psychiatric health conditions, with 1091 of 1427 respondents (76.5%) approving, 132 (9.3%) being uncertain, and only 204 (14.3%) disapproving.

In contrast, among 1427 respondents in sample 1, approval was lower for embryo selection based on physical traits (432 [30.3%]) and behavioral traits (514 [36.0%]). The physical trait with the lowest approval for screening was skin color (824 disapproved [57.7%]), yet 603 respondents (42.3%) were either uncertain (326 [22.8%]) or approved (277 [19.4%]) screening for it. The behavioral trait with the highest approval for screening was intelligence, with 849 respondents (59.5%) either approving (526 [36.9%]) or being uncertain (323 [22.6%]). As preregistered, we conducted a paired samples *t* test to demonstrate that the mean approval rating (measured on a 5-point Likert scale) across the 12 traits (mean [SD], 2.68 [1.10]) was significantly lower than the mean approval rating across the 12 conditions (mean [SD], 3.79 [1.08]; *d* = −1.02; *P* < .001).

#### Potential Concerns Regarding PES

**Figure 4.**
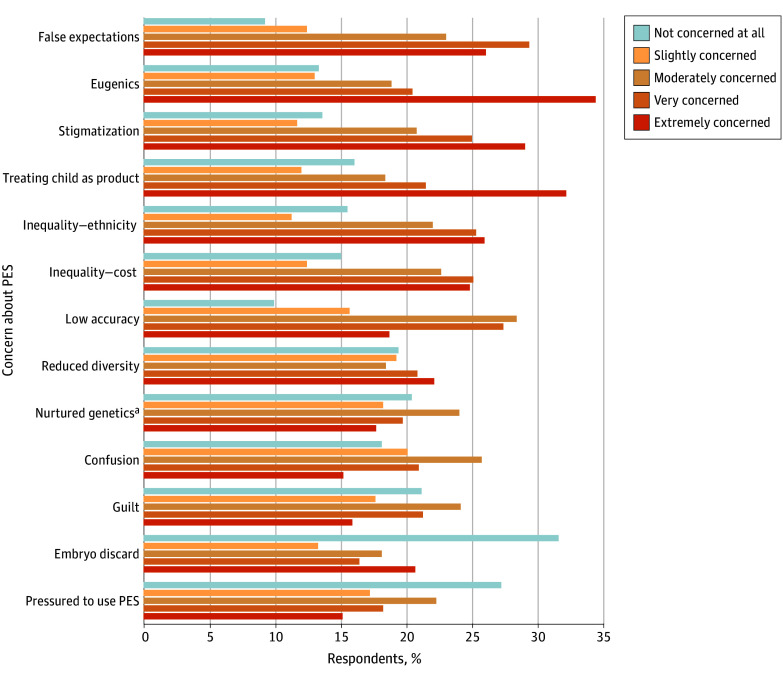
Potential Concerns About Polygenic Embryo Screening (PES) Participants were asked the following: “How concerned, if at all, are you with the following 13 potential concerns that have been raised about polygenic embryo screening (PES)?” They responded using a 5-point Likert scale. The 13 concerns presented are ranked by mean approval (in ascending order). The number of respondents varied from 1420 to 1424 across concern items. ^a^*Nurtured genetics* refers to the act of selectively exposing a child to a particular environment on the basis of a polygenic score—an act that may have a greater influence on the expression of their behavioral traits than their initial genetic predisposition for those behavioral traits.^24^

As demonstrated in Figure 4, participants were asked: “How concerned, if at all, are you with the following 13 potential concerns that have been raised about polygenic embryo screening (PES)?” The proportion of participants who were at least slightly concerned ranged from 68.5% (n = 975 of 1424) to 92.1% (n = 131 of 1422) for all 13 concerns. The proportion of participants who were very to extremely concerned ranged from 49.9% (n = 710 of 1423) to 55.3% (n = 787 of 1422) for the following concerns: parents having false expectations about the future child, promoting eugenic thinking or practices, stigmatizing certain traits and conditions viewed as less desirable, treating embryos like a product by selecting them based on preferred genetic chances for conditions or traits, not being applied equally to all ethnicities, and increasing inequality due to its high costs. Overall, other than having false expectations, respondents appeared less concerned about potential harm to themselves as patients compared with harm to the future child or society.

#### PES Approval by Parenting Status, Interest, and Age

We analyzed the association of PES approval with parenthood status and interest in having children. Parenthood status, with 707 participants having no children (mean [SD], 3.81 [1.05]) and 715 participants having at least 1 child (mean [SD], 3.98 [0.93]), was not associated with approval (*d* = 0.09; *P* = .09). However, interest in having children, with 986 participants not at all interested (mean [SD], 3.81 [1.05]) and 439 participants being at least slightly interested (mean [SD], 3.98 [0.93]), had a significant effect size (*d* = −0.17; *P =* .003), with those less interested in children being less approving of PES. Age, with 460 participants aged 35 years or younger (mean [SD], 4.01 [0.94]) and 967 aged 36 years or older (mean [SD], 3.79 [1.05]), also had a significant effect size (*d* = −0.215; *P* < .001), with older participants being less approving of PES.

### Sample 2: Comparison of Concerns Presented First vs Last

In sample 2 (n = 192), experimentally randomizing the presentation of potential concerns was associated with a significant decrease in general PES approval among the concerns-last group (mean [SD], 3.90 [0.94]) and the concerns-first group (mean [SD], 3.36 [0.87]; *P* < .001; *d* = 0.59) (eFigure 1 and eTables 4 and 5 in Supplement 1). The presentation of concerns first vs last (n = 95 in sample 2a vs 97 in sample 2b) was associated with lower general approval, decreasing 28 percentage points from 71.1% (n = 69 of 97) to 43.2% (n = 41 of 95); uncertainty (neither approving nor disapproving), increasing 24 percentage points from 18.6% (n = 18 of 97) to 43.2% (n = 41 of 95); and higher general disapproval, increasing 4 percentage points from 10.3% (n = 10 of 97) to 13.7% (n = 13 of 95). Participant interest was lower for those presented with concerns first vs last (mean [SD], 2.78 [1.29] vs 3.53 [1.47]; *d* = 0.54; *P* < .001). No significant differences in endorsement were detected on a concern index (averaging across the 13 concerns) between those presented with concerns last vs first (mean [SD], 3.11 [0.77] vs 2.97 [0.80]; *d* = 0.18; *P* = .22). Preregistered bayesian equivalence test results are presented in eFigure 2 and eTable 6 in Supplement 1. All other *t* test results and figures comparing concerns first vs last are reported in eFigure 1 and eTables 4 and 5 in Supplement 1.

## Discussion

In this survey study of the US public, 72.0% of respondents approved of using PES. When the survey specified that the purpose of PES was for embryo selection, most respondents approved of PES use for screening for physical health conditions (77.7%) and psychiatric health conditions (72.0%). In contrast, approval was lower for embryo selection based on behavioral traits (36.0%) and physical traits (30.3%). When the survey specified that the purpose of PES was for parents to prepare for their future child, approval increased to 84.0% for physical health conditions, 80.6% for psychiatric health conditions, 47.0% for behavioral traits, and 41.8% for physical traits. The high level of public approval and interest regarding PES raises concerns over the lack of regulation and professional guidance on what type of polygenic embryo health conditions or traits should be screened and under what circumstances.

When presented with potential concerns, 92.1% of respondents in this study expressed being at least slightly concerned, and 54.8% and 55.3% reported being very and extremely concerned about PES promoting eugenic practices or leading parents to have false expectations about their future child. Further investigation into respondent conceptions of eugenics is warranted, considering that this concern appears to be at odds with the high approval of screening embryos for health conditions for purposes of selection.

In sample 2, we experimentally investigated the association between respondent attitudes toward PES and presenting the list of potential concerns at the start of the survey (vs at the end). Notably, we observed that when concerns were presented at the start of the survey, general approval decreased by 28 percentage points while uncertainty increased by 24 percentage points, but disapproval was associated with an increase of only 4 percentage points. These findings suggest that although respondents shared concerns related to PES (as raised by REIs and patients undergoing IVF), they may not be intuitively salient, as their attitudes were associated with lower approval when concerns were presented at survey onset.

Overall, these findings highlight the urgent need for further research to comprehensively inform the public, patients undergoing IVF, REIs, and genetic counselors on the potential benefits, limitations, and concerns that surround the use of PES. Given the divide in approval between the public and patients undergoing IVF on the one hand and health care professionals on the other,^23,26,35,36,37,38^ we recommend that stakeholder groups, particularly working groups from professional societies, clinicians, and policymakers, consider the basis of this divide. Our study findings suggest high public approval for PES, contrasting with previous concerns voiced by researchers, clinicians, and professional organizations. Our findings underscore the need for immediate attention to regulatory discussions and the formulation of professional guidelines. For example, the American College of Medical Genetics has stated that PES is not yet appropriate for clinical use.^39^ In the meantime, researchers should aim to further elucidate the reasons behind the apparent disparity between public opinion and expert concerns. Our randomized experiment (sample 2) focused on reducing this gap by presenting potential concerns to the public. The presentation of these concerns was associated with a notable decrease in approval; however, it was associated only with a minor increase in disapproval. These findings suggest a nuanced response that warrants further investigation. Subsequent research should clarify to the public why these concerns are pertinent. Additionally, study participants perceived greater benefits than harms from PES, which suggests that it is crucial to also examine the role of values (eg, reproductive freedom and autonomy) and how they might explain the gap in approval between the public and experts. These findings pave the way for important future research directions aimed at bridging the gap between public opinion and expert recommendations. We propose to prioritize 2 efforts: (1) maximize public knowledge and understanding of polygenic scores and their assumptions along with their limitations and (2) investigate the set of values driving perceived benefits of PES.

### Limitations

The survey used stratified sampling limited only to nationally representative quotas for age, gender, and race and ethnicity, which has the potential to skew the participant demographics (eg, as observed with political ideology) and may provide less accurate attitudes of the general US public. However, as reported in eTable 1 in Supplement 1, weighted analyses based on the US population distribution on political ideology gave similar levels of approval.

The observed decrease in PES approval associated with presenting potential concerns first may be due, in part, to social desirability bias, whereby respondents answered in a way they believed was socially acceptable. At the same time, the slight increase in disapproval when concerns were presented first might be amplified if individuals were provided with more elaborate explanations of the potential concerns. Future research should aim to further investigate factors that may help narrow the gap between public and stakeholder attitudes toward PES.

## Conclusions

The findings of this survey study shed light on the US public perspective on PES and highlight the importance of comprehensively informing both the public and health care professionals about their varying perspectives. Our results suggest that efforts are needed to comprehensively inform the public, patients undergoing IVF, REIs, and genetic counselors on the limitations and concerns that surround the use of PES. We further recommend that stakeholder groups, particularly working groups from professional societies, clinicians, and policymakers, consider the basis of this gap between the attitudes of health care professionals and the attitudes of the public.

## Notes

Supplement 1. **eMethods 1.** Preregistrations **eMethods 2.** Survey Materials and Data Availability **eMethods 3.** Generation of Embryo Risk Scores for Introduction **eTable 1.** Sample 1: Political Ideology Weighted Sample on Polygenic Embryo Screening Approval Distribution **eTable 2.** Sample 1: Income Weighted Sample on Polygenic Embryo Screening Approval Distribution **eTable 3.** Sample 1: Education Weighted Sample on Polygenic Embryo Screening Approval Distribution **eAppendix.** Sample 1 and Sample 2a: Comparison of Approval, Interest, and Concerns **eFigure 1.** Sample 2: Concerns Presented Last vs Concerns Presented First **eFigure 2.** Sample 2: Concerns First vs Concerns Last—Equivalence Bayesian *t* Test on Concerns Plot **eTable 4.** Sample 2: Concerns First vs Concerns Last—Descriptive Statistics **eTable 5.** Sample 2: Concerns First vs Concerns Last—Independent Samples *t* Test **eTable 6.** Sample 2: Concerns First vs Concerns Last—Equivalence Bayesian *t* Test on Concerns
eReferences.

Supplement 2.
Data Sharing Statement
