# Supplementary material for: Public Attitudes, Interests, and Concerns Regarding Polygenic Embryo Screening
Source: JAMA Netw Open. 2024 May 14;7(5):e2410832. doi: 10.1001/jamanetworkopen.2024.10832 (PMC11094562; doi:10.1001/jamanetworkopen.2024.10832)
Supplement: Supplement 1. — eMethods 1. Preregistrations eMethods 2. Survey Materials and Data Availability eMethods 3. Generation of Embryo Risk Scores for Introduction eTable 1. Sample 1: Political Ideology Weighted Sample on Polygenic Embryo Screening Approval Distribution eTable 2. Sample 1: Income Weighted Sample on Polygenic Embryo Screening Approval Distribution eTable 3. Sample 1: Education Weighted Sample on Polygenic Embryo Screening Approval Distribution eAppendix. Sample 1 and Sample 2a: Comparison of Approval, Interest, and Concerns eFigure 1. Sample 2: Concerns Presented Last vs Concerns Presented First eFigure 2. Sample 2: Concerns First vs Concerns Last—Equivalence Bayesian t Test on Concerns Plot eTable 4. Sample 2: Concerns First vs Concerns Last—Descriptive Statistics eTable 5. Sample 2: Concerns First vs Concerns Last—Independent Samples t Test eTable 6. Sample 2: Concerns First vs Concerns Last—Equivalence Bayesian t Test on Concerns eReferences. [file jamanetwopen-e2410832-s001.pdf]

Supplementary Online Content

Furrer RA, Barlevy D, Pereira S, Carmi S, Lencz T, Lázaro-Muñoz G. Public attitudes, interests, and concerns regarding polygenic embryo screening. *JAMA Netw Open*. 2024;7(5):e2410832. doi:10.1001/jamanetworkopen.2024.10832

eMethods 1. Preregistrations

eMethods 2. Survey Materials and Data Availability

eMethods 3. Generation of Embryo Risk Scores for Introduction

eTable 1. Sample 1: Political Ideology Weighted Sample on Polygenic Embryo Screening Approval Distribution

eTable 2. Sample 1: Income Weighted Sample on Polygenic Embryo Screening Approval Distribution

eTable 3. Sample 1: Education Weighted Sample on Polygenic Embryo Screening Approval Distribution

eAppendix. Sample 1 and Sample 2a: Comparison of Approval, Interest, and Concerns

eFigure 1. Sample 2: Concerns Presented Last vs Concerns Presented First

eFigure 2. Sample 2: Concerns First vs Concerns Last—Equivalence Bayesian *t* Test on Concerns Plot

eTable 4. Sample 2: Concerns First vs Concerns Last—Descriptive Statistics

eTable 5. Sample 2: Concerns First vs Concerns Last—Independent Samples *t* Test

eTable 6. Sample 2: Concerns First vs Concerns Last—Equivalence Bayesian *t* Test on Concerns

This supplementary material has been provided by the authors to give readers additional information about their work.

© 2024 Furrer RA et al. *JAMA Network Open.*

eMethods 1. Preregistrations

The survey preregistrations are uploaded at the following links:

Sample1: https://aspredicted.org/JSJ_6BR
Sample2: https://aspredicted.org/LM7_FZP

eMethods 2. Survey Materials and Data Availability

The survey materials are uploaded at the following links:
Sample 1: https://researchbox.org/1646&PEER_REVIEW_passcode=XAFEJF
Sample2: https://researchbox.org/1370&PEER_REVIEW_passcode=GLTTIB

eMethods 3. Generation of Embryo Risk Scores for Introduction

As part of the introduction, we informed participants about PES by presenting two embryos that varied in their genetic risk estimates across four conditions (see image below). Note that each risk/chance value was explained to participants in additional materials.

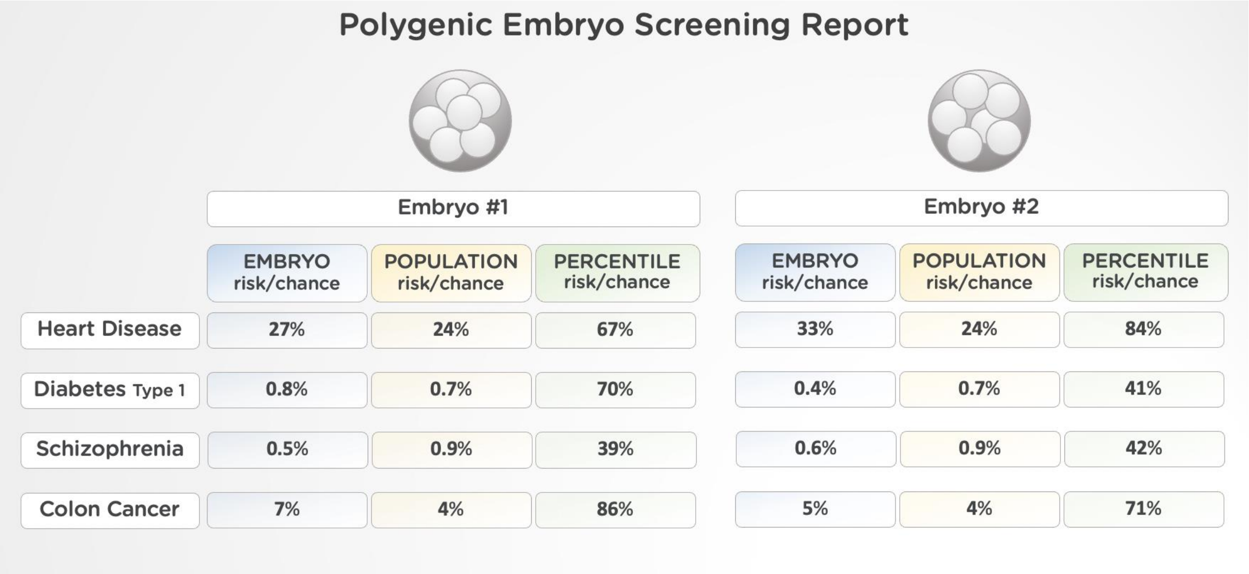

Polygenic Embryo Screening Report

	Embryo #1			Embryo #2		
	EMBRYO risk/chance	POPULATION risk/chance	PERCENTILE risk/chance	EMBRYO risk/chance	POPULATION risk/chance	PERCENTILE risk/chance
Heart Disease	27%	24%	67%	33%	24%	84%
Diabetes Type 1	0.8%	0.7%	70%	0.4%	0.7%	41%
Schizophrenia	0.5%	0.9%	39%	0.6%	0.9%	42%
Colon Cancer	7%	4%	86%	5%	4%	71%

The difference between the standardized polygenic risk scores of the two embryos was computed based on their risk percentiles q1 and q2 using the following command in R: qnorm(q2/100) - qnorm(q1/100). For sibling embryos, the difference is expected to be distributed as a standard normal random variable. Thus, it would be rare for this difference to exceed 2 (or be less than -2). A difference within [-2,2] is feasible, but most of the time it would be smaller (i.e., [-1,1] or [-0.5,0.5]).

We used the following inputs (K=; r=; q=) to compute each embryo's risk in R:

q= percentile risk/chance

- Heart Disease: q1=67; q2=84

© 2024 Furrer RA et al. *JAMA Network Open.*

qnorm(q2/100) - qnorm(q1/100)= 0.55

- Type 1 Diabetes: q1=70; q2=41
  qnorm(q2/100) - qnorm(q1/100)= -0.75

- Schizophrenia: q1=39; q2=42
  qnorm(q2/100) - qnorm(q1/100)= 0.08

- Colon Cancer: q1=86; q2=71
  qnorm(q2/100) - qnorm(q1/100)= -0.52

K= Population risk/chance (lifetime risk)[1,2,3,4]

- Heart Disease: K=0.24
- Type 1 Diabetes: K=0.007
- Schizophrenia: K=0.009
- Colon Cancer: K=0.04

R^2= Variance explained by PRS

R^2 is the proportion of variance in liability explained by the PRS. We used R^2= 0.08 for all conditions as an upper bound on the current PRS effect size. This is based on values of 6% for Crohn's disease[5], 7% for schizophrenia[6] and 9% for type 2 diabetes[7]

Embryo Risk/Chance Output:

We used the liability threshold model[5]. Given prevalence K, PRS percentile q, and proportion of variance explained r2, we used the following R code to compute the risk.

- zK=qnorm(1-K); s=qnorm(q/100,0,sqrt(r2)); risk=pnorm((zK-s)/sqrt(1-r2),lower.tail = F)

© 2024 Furrer RA et al. *JAMA Network Open.*

eTable 1. Sample 1: Political Ideology Weighted Sample on Polygenic Embryo Screening Approval Distribution

eTable 1a

**Political Ideology**	**Prolific Sample**	**Gallup (July, 2023)**	**Weights**
Conservative	25%	27%	1.08
Moderate	18%	45%	2.5
Liberal	57%	25%	0.44

Note: We used Gallup's[8] data on political ideology from July 2023 (the month we ran the study) to calculate weights and re-analyzed approval with a weighted sample that matches the U.S. population distribution on political ideology. We measured political ideology on a scale from 1-7 and then aggregated values 1-3 to identify liberals, a value of 4 to identify moderates, and values 5-7 for conservatives.

eTable 1b

**Polygenic Embryo Screening Approval Distribution**	Prolific Sample **weighted** (n=1382)	Prolific Sample **unweighted** (n=1427)
Strongly disapprove	4.20%	3.36%
Disapprove	7.90%	7.42%
Neither approve nor disapprove	20.10%	17.24%
Approve	42.10%	43.45%
Strongly approve	25.80%	28.52%

© 2024 Furrer RA et al. *JAMA Network Open.*

eTable 2. Sample 1: Income Weighted Sample on Polygenic Embryo Screening Approval Distribution

eTable 2a

Income	Census 2022	Prolific Sample	Weights
$0–$49,999	34%	42%	0.812
≥ $50,000–109,999	32.8%	39%	0.845
≥ $110,000	33.2%	19%	1.71

Note: Weights were calculated using US census data[9]

eTable 2b

Polygenic Embryo Screening Approval Distribution	Prolific Sample **weighted** (n=789)	Prolific Sample **unweighted** (n=1427)
Strongly disapprove	3.9%	3.36%
Disapprove	7.5%	7.42%
Neither approve nor disapprove	18.6%	17.24%
Approve	41.2%	43.45%
Strongly approve	28.7%	28.52%

© 2024 Furrer RA et al. *JAMA Network Open.*

eTable 3. Sample 1: Education Weighted Sample on Polygenic Embryo Screening Approval Distribution

eTable 3a

Education	US Census (2023)	Prolific sample	Weights
less than a high school diploma	9%	0.70%	12.857
high school diploma	28%	12%	2.333
some college	15%	21%	0.714
associate's degree	10%	11%	0.909
bachelor's degree	23%	38.10%	0.603
Post-college degree	14%	16.60%	0.843

Note: Weights were calculated using US census data[10]

eTable 3b

**Polygenic Embryo Screening Approval Distribution**	Prolific Sample **weighted** (n=1419)	Prolific Sample **unweighted** (n=1427)
Strongly disapprove	4.0%	3.36%
Disapprove	6.5%	7.42%
Neither approve nor disapprove	17.3%	17.24%
Approve	43.0%	43.45%
Strongly approve	29.3%	28.52%

© 2024 Furrer RA et al. *JAMA Network Open.*

eAppendix. Sample 1 and Sample 2a: Comparison of Approval, Interest, and Concerns

Sample 1 (n=1427) was recruited by the sampling firm prolific and stratified to be nationally representative on the basis of gender, race/ethnicity and age. Sample 2a (n=97) had the concerns presented last (at the end of the survey) and was therefore the same survey as sample 1. Sample 2a was also recruited from prolific, but did not specify a nationally representative quota for gender, race/ethnicity and age. We compared mean approval, interest and concerns between sample 1 and sample 2a using a series of Welch's t-tests.

Results demonstrate that no significant differences in PES approval were observed between sample 1 (*M*=3.86, *SD*=1.02) and sample 2a (*M*=3.90, *SD*=0.941), *t*(111.90)=-.40, *p*=0.69; *d*=.04. Significant differences in PES interest were observed between sample 1 (*M*=3.13, *SD*=1.38) and sample 2a (*M*=3.53, *SD*=1.47), *t*(107.82)=-2.6, *p*=0.005; *d*=.29. No significant differences in PES concerns were observed between sample 1 (*M*=3.18, *SD*=0.95) and sample 2a (*M*=3.11, *SD*=0.77), *t*(116.58)=0.85, *p*=0.40; *d*=.08. We report these similarities and differences between the two samples for the sake of transparency, however, given the large differences in sample sizes and sampling methods, these results should not be overly interpreted as suggesting that there are either meaningful differences or a lack of differences between the two samples.

© 2024 Furrer RA et al. *JAMA Network Open.*

eFigure 1. Sample 2: Concerns Presented Last vs Concerns Presented First

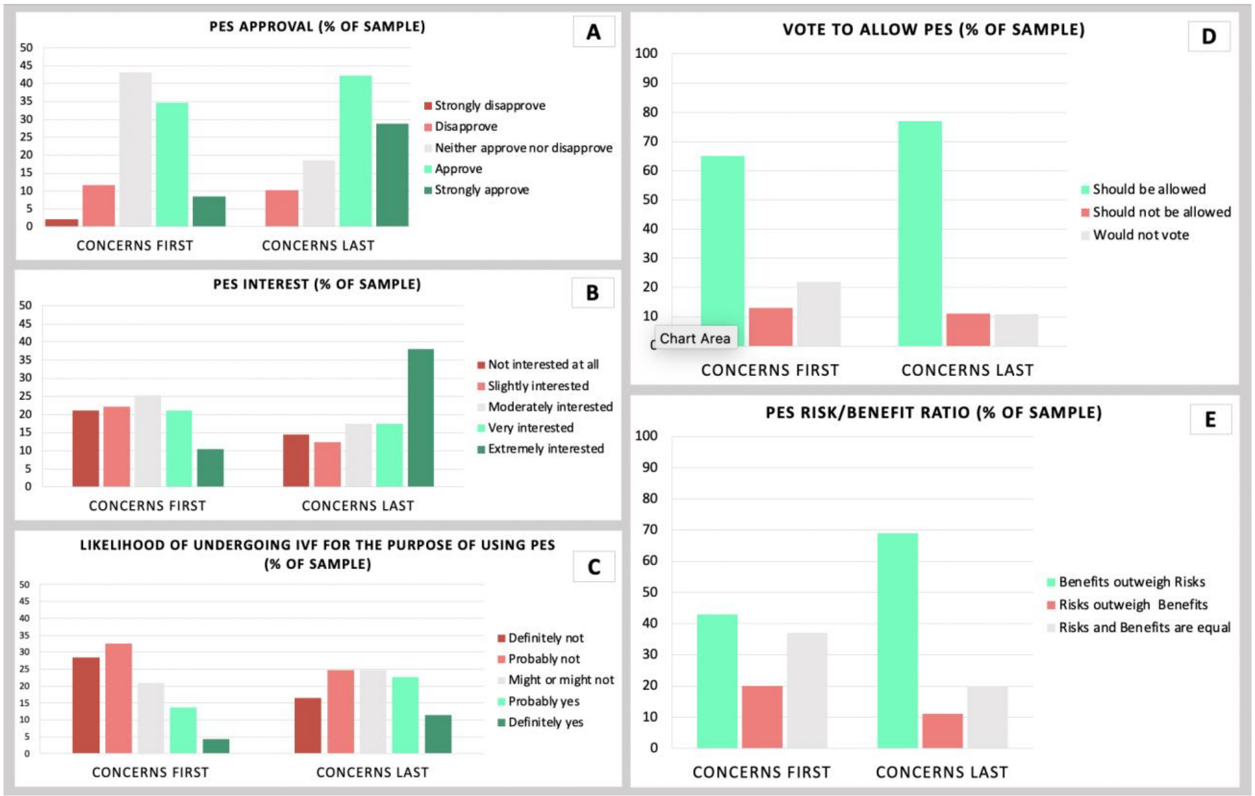

© 2024 Furrer RA et al. *JAMA Network Open.*

eFigure 2. Sample 2: Concerns First vs Concerns Last—Equivalence Bayesian *t* Test on Concerns Plot

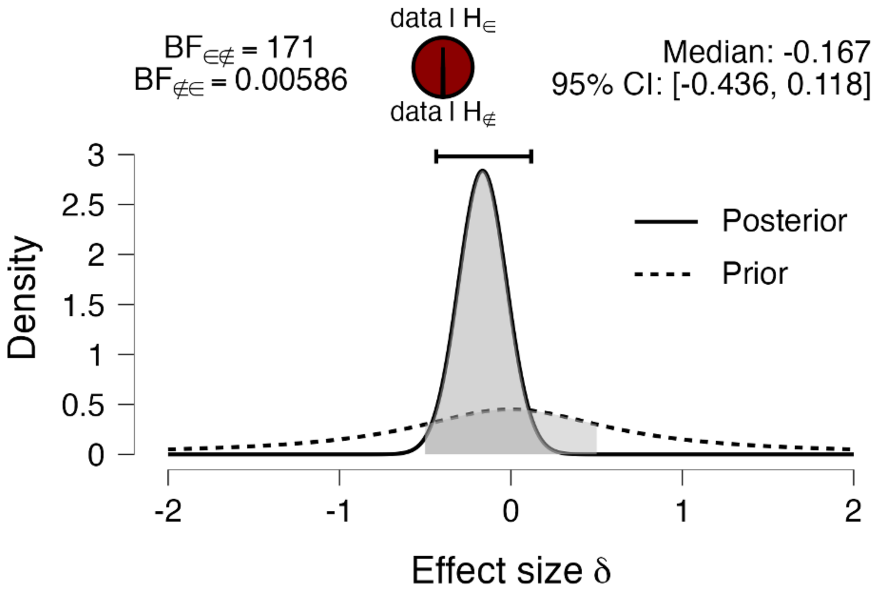

© 2024 Furrer RA et al. *JAMA Network Open.*

eTable 4. Sample 2: Concerns First vs Concerns Last—Descriptive Statistics

	Group	N	Mean	SD	SE
Concerns (AVG)	First	95	2.973	0.804	0.082
	Last	97	3.113	0.772	0.078
Conditions Approval (AVG)	First	95	3.478	1.124	0.115
	Last	97	4.003	0.941	0.095
Traits Approval (AVG)	First	95	2.347	1.137	0.117
	Last	97	2.767	1.104	0.112
Information Purpose (AVG)	First	95	0.758	2.538	0.260
	Last	97	1.732	2.303	0.234
Preparedness Purpose (AVG)	First	95	1.158	2.349	0.241
	Last	97	2.103	2.172	0.221
Selection Purpose (AVG)	First	95	0.053	2.647	0.272
	Last	97	1.113	2.483	0.252
Family Selection Purpose (AVG)	First	95	0.284	2.495	0.256
	Last	97	1.237	2.401	0.244
IVF Approval	First	95	3.811	0.903	0.093
	Last	97	4.196	0.772	0.078
PES Approval	First	95	3.358	0.874	0.090
	Last	97	3.897	0.941	0.096
Estimate approval for General Population	First	95	3.200	0.858	0.088
	Last	97	3.454	0.764	0.078
PES Interest	First	95	2.779	1.290	0.132
	Last	97	3.526	1.466	0.149
IVF Interest for PES	First	95	2.326	1.153	0.118
	Last	97	2.876	1.260	0.128

Note: Group name “First” refers to participants from sample 2a randomly assigned to being presented with concerns first. Group name “Last” refers to participants from sample 2b randomly assigned to being presented with concerns first. (AVG)= Average of items.

© 2024 Furrer RA et al. *JAMA Network Open.*

eTable 5. Sample 2: Concerns First vs Concerns Last—Independent Samples *t* Test

	t	df	p	Cohen's d	SE Cohen's d
Concerns (AVG)	-1.237	190	0.218	-0.179	0.145
Conditions (AVG)	-3.510	190	< .001	-0.507	0.149
Traits (AVG)	-2.596	190	0.010	-0.375	0.147
Informational Purpose (AVG)	-2.786	190	0.006	-0.402	0.147
Preparedness Purpose (AVG)	-2.896	190	0.004	-0.418	0.147
Selection Purpose (AVG)	-2.865	190	0.005	-0.413	0.147
Family Selection Purpose (AVG)	-2.696	190	0.008	-0.389	0.147
IVF Approval	-3.180	190	0.002	-0.459	0.148
PES Approval	-4.111	190	< .001	-0.593	0.151
Estimate Approval for General Population	-2.164	190	0.032	-0.312	0.146
PES Interest	-3.746	190	< .001	-0.541	0.150
Estimate Interest for General Population	-0.744	190	0.458	-0.107	0.145
IVF Interest for PES	-3.153	190	0.002	-0.455	0.148

© 2024 Furrer RA et al. *JAMA Network Open.*

eTable 6. Sample 2: Concerns First vs Concerns Last—Equivalence Bayesian *t* Test on Concerns

	Model Comparison	BF	error %
Concern_AVG	$\delta \in I$ vs. H_1	2.529	1.835×10^{-5}
	$\delta \notin I$ vs. H_1	0.015	0.003
	$\delta \in I$ vs. $\delta \notin I$	170.771	5.436×10^{-7}
	$\delta \notin I$ vs. $\delta \in I$	0.006	0.016

Note. As pre-registered, given that we did not find a significant difference in the average concerns between participants presented with concerns first (sample 2a) vs. last (sample 2b), we conducted exploratory Bayesian Interval-Null testing (equivalence testing) to determine how much evidence our data provide in favor of the null. Specifically, we tested whether the order of concerns (presented first vs. last) had no effect on the overall average of the concerns themselves. We set our equivalence region to -0.5 – 0.5 and used the default Cauchy prior with a scale of 0.707. We tested the degree to which the data support the hypothesis that the parameter lies inside versus outside the equivalence region and found that the non-overlapping-hypothesis Bayes factor in favor of the interval-null was 171. Overall, these results demonstrate strong evidence for the null hypothesis when comparing participants in the concerns first condition with those in the concerns last condition.

© 2024 Furrer RA et al. *JAMA Network Open.*

eReferences.

1. Heart disease prevalence - health, United States. Centers for Disease Control and Prevention. Accessed October 14, 2023. https://www.cdc.gov/nchs/hus/topics/heart-disease-prevalence.htm.

2. Prevalence of diagnosed diabetes. Centers for Disease Control and Prevention. September 30, 2022. Accessed October 14, 2023. https://www.cdc.gov/diabetes/data/statistics-report/diagnosed-diabetes.html.

3. Perälä J, Suvisaari J, Saarni SI, et al. Lifetime Prevalence of Psychotic and Bipolar I Disorders in a General Population. *Arch Gen Psychiatry.* 2007;64(1):19–28. doi:10.1001/archpsyc.64.1.19

4. Cancer of the colon and rectum - cancer stat facts. SEER. Accessed October 14, 2023. https://seer.cancer.gov/statfacts/html/colorect.html.

5. Lencz T, Backenroth D, Granot-Hershkovitz E, et al. Utility of polygenic embryo screening for disease depends on the selection strategy. *Elife*. 2021;10:e64716. Published 2021 Oct 12. doi:10.7554/eLife.64716

6. Trubetskoy, V., Pardiñas, A.F., Qi, T. *et al.* Mapping genomic loci implicates genes and synaptic biology in schizophrenia. *Nature* 604, 502–508 (2022). https://doi.org/10.1038/s41586-022-04434-5

7. Ge, T., Irvin, M.R., Patki, A. *et al.* Development and validation of a trans-ancestry polygenic risk score for type 2 diabetes in diverse populations. *Genome Med* 14, 70 (2022). https://doi.org/10.1186/s13073-022-01074-2

8. Party Affiliation. Gallup.com. September 20, 2007. Accessed February 24, 2024. https://news.gallup.com/poll/15370/party-affiliation.aspx

9. HINC-06. Income Distribution to $250,000 or More for Households. Census.gov. Accessed February 24, 2024. https://www.census.gov/data/tables/time-series/demo/income-poverty/cps-hinc/hinc-06.html

10. Census Bureau Releases New Educational Attainment Data. Census.gov. Accessed February 24, 2024. https://www.census.gov/newsroom/press-releases/2023/educational-attainment-data.html

© 2024 Furrer RA et al. *JAMA Network Open.*
