# Supplementary material for: Public Attitudes, Interests, and Concerns Regarding Polygenic Embryo Screening
Source: JAMA Netw Open. 2024 May 14;7(5):e2410832. doi: 10.1001/jamanetworkopen.2024.10832 (PMC11094562; doi:10.1001/jamanetworkopen.2024.10832)
Supplement: Supplement 2. — Data Sharing Statement [file jamanetwopen-e2410832-s002.pdf]

Data Sharing Statement

Furrer. Public Attitudes, Interests, and Concerns Regarding Polygenic Embryo Screening. *JAMA Netw Open*. Published May 14, 2024. doi:10.1001/jamanetworkopen.2024.10832

Data
Data available: Yes
Data types: Deidentified participant data, Other (please specify)
Additional Information: Deidentified data will be made available under reasonable request given that some new analyses of this data may be used for additional manuscripts.
How to access data: requests can be made to remy_furrer@hms.harvard.edu
When available: With publication

Supporting Documents
Document types: Statistical/analytic code, Informed consent form, Other (please specify)
Additional Information: Survey Questions are uploaded on researchbox.
How to access documents: https://researchbox.org/1646&PEER_REVIEW_passcode=XAFEJF (sample 1) and https://researchbox.org/1370&PEER_REVIEW_passcode=GLTTIB (sample 2)
When available: With publication

Additional Information
Who can access the data: researchers affiliated with a university whose proposed use has been approved.
Types of analyses: non-commercial purpose
Mechanisms of data availability: with investigator support
